# Three Fluoroscopy-Guided Epidural Blood Patches for the Management of Spontaneous Intracranial Hypotension

**DOI:** 10.7759/cureus.78578

**Published:** 2025-02-05

**Authors:** Mitsutaka Edanaga, Sayaka Morohara, Kosuke Hamada, Katsuya Komatsu, Michiaki Yamakage

**Affiliations:** 1 Anesthesiology, Sapporo Medical University, Sapporo, JPN; 2 Anesthesiology, Sapporo Kiyota Hospital, Sapporo, JPN; 3 Anesthesiology, Rishinkai Orthopedic Hospital, Sapporo, JPN; 4 Anesthesiology, Sapporo Medical University School of Medicine, Sapporo, JPN; 5 Neurosurgery, Sapporo Medical University School of Medicine, Sapporo, JPN

**Keywords:** epidural blood patch, fluoroscopy-guided, migraine headache, prone position, spontaneous intracranial hypotension

## Abstract

This report describes the use of three fluoroscopy-guided epidural blood patch procedures to treat a patient with spontaneous intracranial hypotension. A 42-year-old woman with no history of history of surgery or trauma presented with headache and dizziness. Magnetic resonance imaging revealed an extradural cerebrospinal fluid leak collection leading to a diagnosis of spontaneous intracranial hypotension. Common symptoms of spontaneous intracranial hypotension include orthostatic headache, nausea, neck pain, hearing disturbance, dizziness, and aural fullness. However, the cause of spontaneous intracranial hypotension in this patient was not clear. Conservative therapy as the first treatment for spontaneous intracranial hypotension failed to alleviate the patient's symptoms. Subsequently, the anesthetist performed fluoroscopy-guided preoperative epidural catheterization in the prone position thrice. After the symptomatic improvement, the patient was discharged on the 118^th^ day after admission. Although the treatment with an epidural blood patch became the standard of care in Japan from 2016, the fluoroscopy-guided method has not been generalized yet. Our report suggests that the fluoroscopy-guided approach for the epidural blood patch in the prone position is safe and reliable.

## Introduction

Spontaneous intracranial hypotension (SIH) is a rare neurological disorder that occurs when cerebrospinal fluid (CSF) leaks from the spine. The average incidence of spontaneous intracranial hypotension was 4.3 per 100,000 population (95% CI, 1.9 to 6.7) for women and 2.9 per 100,000 population (95% CI, 0.8 to 5.1) for men, indicating a slight female preponderance. The average age of onset for SIH was 40 years [1,2]. The most common symptoms of spontaneous intracranial hypotension include orthostatic headache, nausea, neck pain, hearing disturbance, dizziness, and aural fullness [3]. The cause of cerebrospinal fluid leak is most commonly a tear in the layer surrounding the spinal cord and brain [4]. However, in some cases, the cause may be unknown. In SIH, CSF collection outside the dura mater can be detected on magnetic resonance imaging (MRI) of the spine [5]. Conservative therapy for SIH involves bed rest, hydration, and caffeine [2,6]. Other effective measures include avoiding Valsalva maneuvers, using an abdominal blinder, and using NSAIDs, opioids, and anti-emetics [6]. When conservative therapy fails to improve the symptoms, an epidural blood patch can be considered for severe cases [7]. In this report, we describe the first case of a female patient with an unidentified CSF leak who achieved symptomatic improvement after three fluoroscopy-guided epidural blood patch procedures. All procedures of the epidural blood patch were performed in the prone position.

## Case presentation

**Figure 1:**
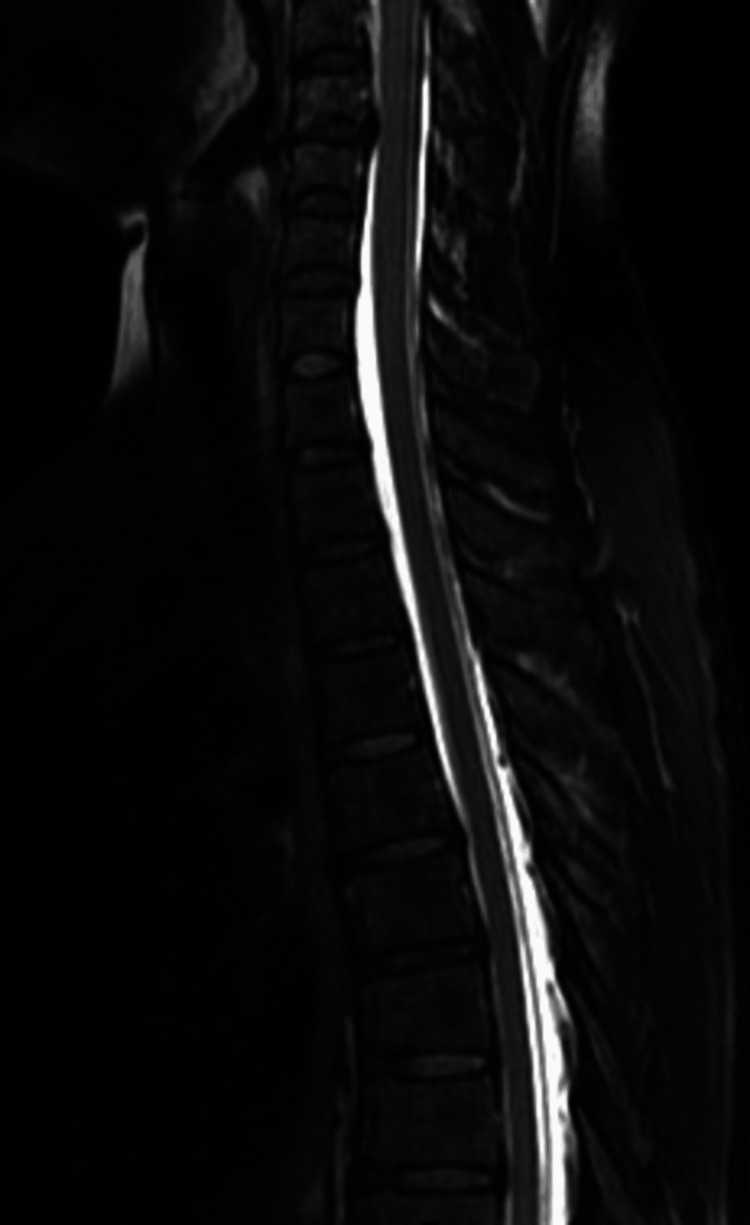
Magnetic resonance imaging acquired at admission. Sagittal T2-weighted imaging shows extensive cerebrospinal fluid collection in the extradural space of the third cervical vertebra to the sacrum. Water signal (white color) in the epidural space and the dural sac was noted.

**Figure 2:**
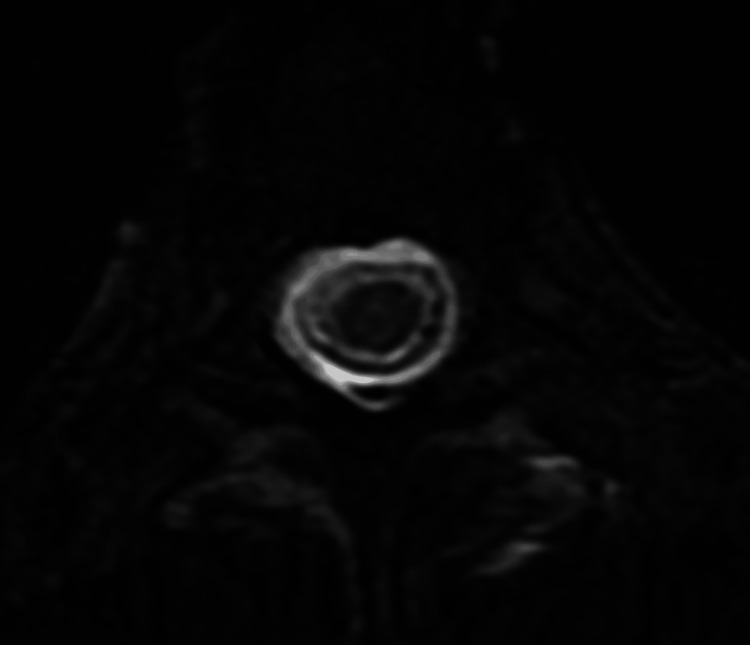
The fat-suppressed T2-weighted axial image at admission. The fat-suppressed T2-weighted axial image shows that the water signal in the epidural space and the dural sac.

A 42-year-old woman was transferred to the neurosurgical ward of Sapporo Medical University from another hospital with complaints of headache and dizziness. She had no previous history of neurosurgery, spinal surgery, or spinal trauma. The sagittal and axial magnetic resonance imaging examination at admission showed extensive cerebrospinal fluid collection into the extradural space from the level of the third cervical vertebra to the sacrum (Figure 1, 2) leading to a diagnosis of SIH. However, the source of the CSF leak could not be identified.

As conservative treatment of hydration, bed rest, and analgesics (acetaminophen, diclofenac sodium, and pregabalin) failed to improve symptoms, we decided to perform an epidural blood patch procedure. Standard pre-treatment laboratory test results for blood clotting function analysis showed a platelet count of 252 × 10^3^/mL, prothrombin time test with an INR (PT-INR) of 1.02, and a activated partial thromboplastin time (APTT) 28.0 s.

Despite the risk of accidental dural puncture and the low success rates for initial epidural patch performed for spontaneous intracranial hypotension, we planned to perform fluoroscopy-guided epidural blood patch [2]. The first epidural blood patch on the 6th day after admission. The patient’s blood pressure, heart rate, and oxygen saturation were recorded.

**Figure 3:**
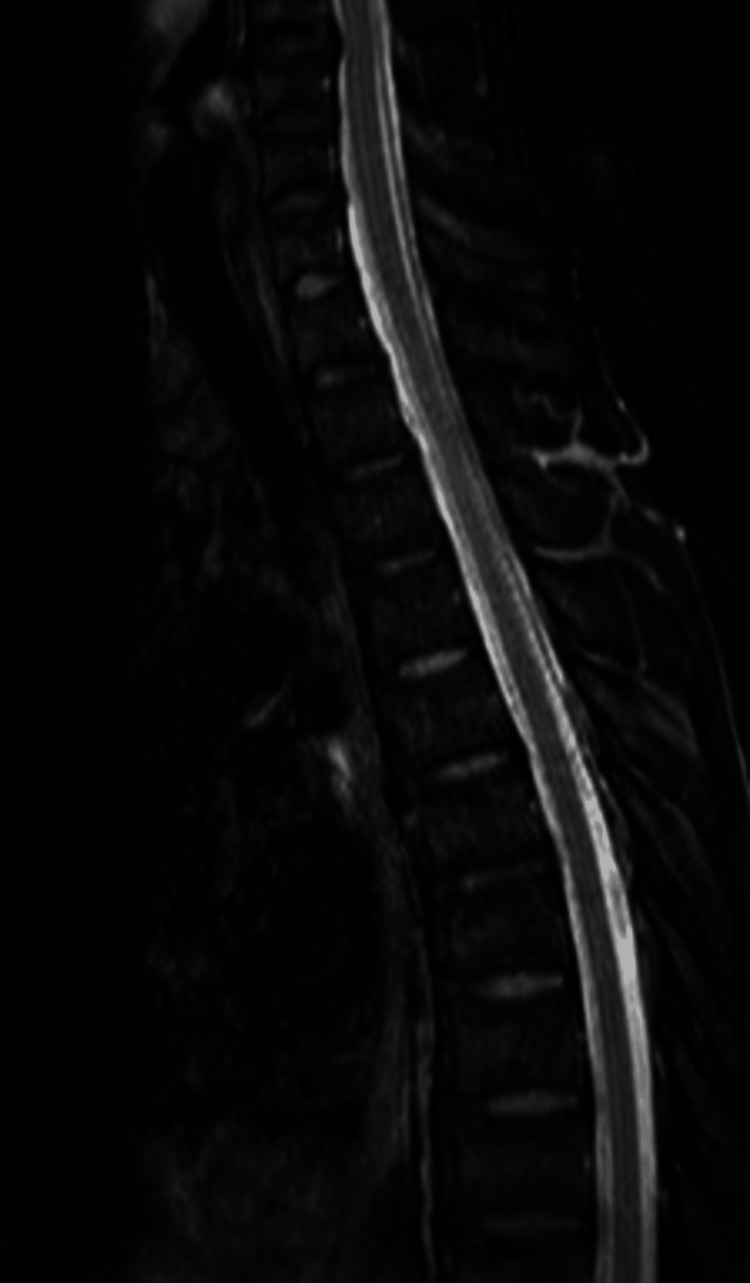
Magnetic resonance imaging after the third epidural blood patch. Sagittal T2-weighted image shows no extradural cerebrospinal fluid collection.

Blood required for injection into the epidural space during an epidural blood patch is usually collected from the vein of the forearm in the anesthesiology outpatient department. However, in our case, radial artery catheterization in the prone position was conducted to collect sufficient blood for the procedure. After consultation with the neurosurgeon, we began the first non-targeted epidural blood patch procedure at the level of L1 and L2. The epidural puncture was performed by using the loss of resistance method by saline and the dorsal epidural space was marked with contrast medium. A total of 40 mL of blood was slowly injected into the epidural space. The leakage from cervical to thoracic level persisted after the first procedure as observed by MRI. The patient was advised bed rest. We performed a second epidural blood patch (confirmed fluoroscopically) at the level of T10 and T11 on day 26 after the first procedure where we injected 40 mL of blood into the thoracic epidural space. Postoperative MRI showed leakage of CSF from above T8, and the patient’s symptoms (headache, dizziness, ambulatory dysfunction) persisted. Thus, we performed a third epidural blood patch on the 14th day after the second procedure. In the third epidural blood patch, we performed the treatment at the level of T8 and T9 using a blood volume of 25 mL, after which no extradural cerebrospinal fluid collection was apparent on postoperative MRI performed on the 7th day after the third procedure (Figure 3). There were no complications following a series of three epidural blood patch procedures. The patient was able to walk normally on the 57th day after the third procedure, and was discharged on the 118th day after admission.

## Discussion

While using an epidural blood patch for SIH is widely reported, this is the first report of three fluoroscopy-guided epidural blood patch procedures used to treat a patient with SIH [2]. Previously published reports showed that the success rate for the first epidural blood patch varied widely, ranging from 13% to 75% [2,3,9,10]. These data were in agreement with the time course of the present patient. This highlights the importance of the approach for multiple epidural blood patch procedures. Ohtonari et al. indicated that the risk for complications caused by multiple epidural blood patches and the practitioner’s stress level increase significantly with the frequency of the epidural blood patch [11]. In terms of safety and certainty, we would like to recommend the fluoroscopy-guided method. While the epidural blood patch became the insurance adaptation in 2016 in Japan, [12] the fluoroscopy-guided method has not been generalized yet. Many anesthesiologists would agree that the epidural puncture at the level of the thoracic region is more difficult than that at the lumbar region. A recent retrospective study has reported that an epidural blood patch under fluoroscopic guidance in SIH was more effective than that with a blind approach [13]. The prone position is essential for a successful fluoroscopy-guided epidural blood patch. The practitioner should consider some important points about the fluoroscopy-guided method. 

**Figure 4:**
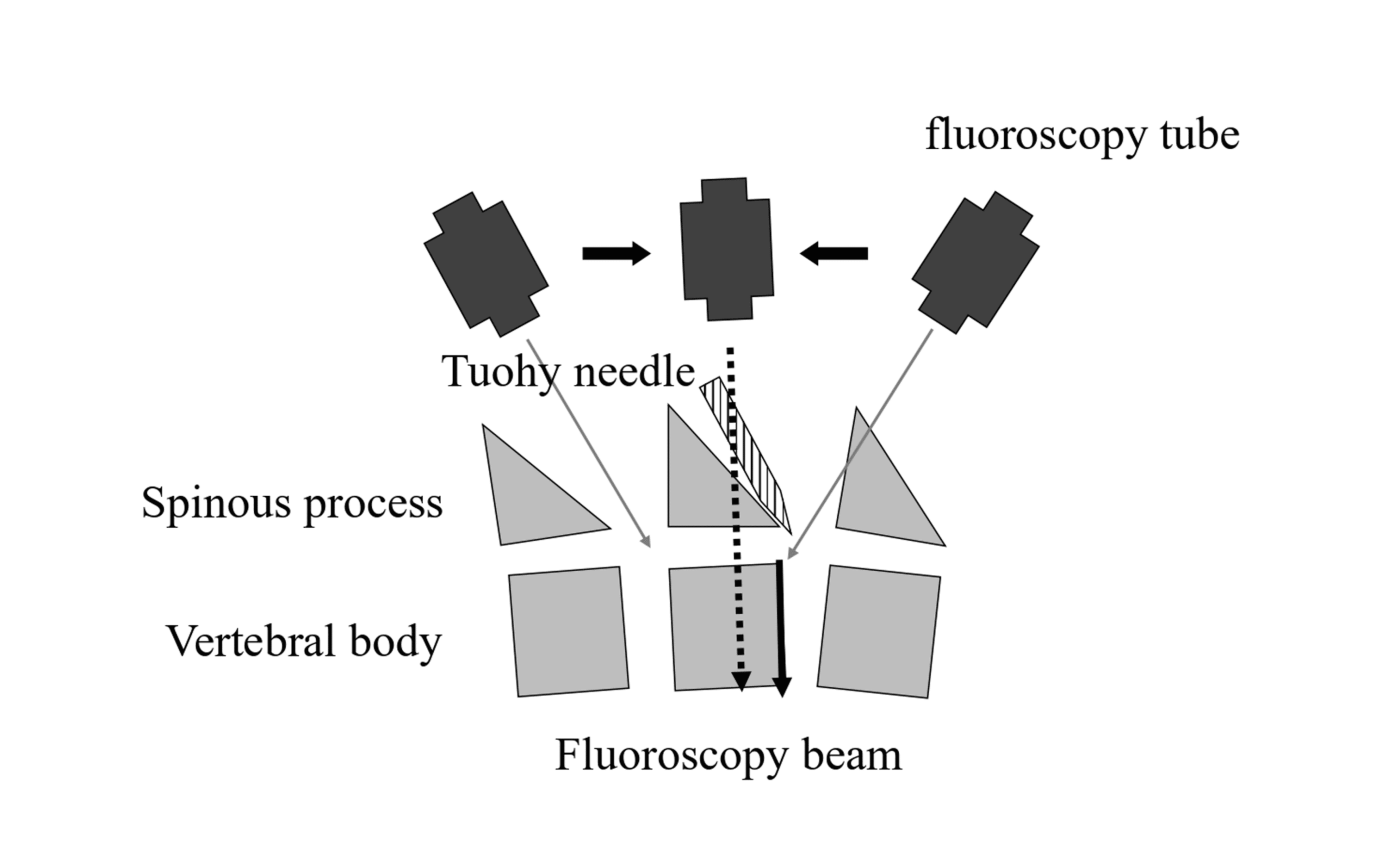
Alignment of the fluoroscopic beam with the superior surface of the vertebral body in the puncture region. It is necessary to align the fluoroscopy beam (dotted arrow) by fluoroscopy tube parallel to the superior surface (black arrow) of the vertebral body in the puncture region.

Fluoroscopic guided-preoperative epidural catheterization is depicted in Figure 3. Firstly, a pillow is placed under the pelvis to increase the gap between the spinous processes. Next, the practitioner has to perform the treatment from the right side of the patient similar to a cardiovascular surgeon performing stent graft insertion or a cardiologist performing transcatheter aortic valve replacement. Thirdly, to visualize the gap between the upper and lower spinous processes, it is necessary to align the fluoroscopy beam (dotted arrow in Figure 4) parallel to the superior surface (black arrow in Figure 4) of the vertebral body in the puncture region. Thus, the Tuohy needle can be easily advanced into the gap between the vertebral bodies under fluoroscopy guidance. When the epidural puncture using the loss of resistance method by saline is successful, the practitioner should confirm that the dorsal epidural space was contrasted with the contrast medium.

Earlier, it was thought that there was no association between injected blood volume of more than 20 mL and the success rate of epidural blood patch procedure [7]. However, a recent report indicates that 20 mL of blood injected in the epidural space would be spread into 4.6 vertebral body intervals [14]. Although we selected 40 mL for non-targeted cerebrospinal fluid leak for the first two epidural blood patch procedures, a recent report has explained that the typical volume was in the range of 30-80 mL. The discussion of appropriate blood volume at the blood patch for spontaneous intracranial hypotension will be necessary in the future. It is also necessary to consider the risk of radiation exposure to the patient when fluoroscopy is used. Accordingly, we reduced the fluoroscopy time as much as possible ensuring that the effect of radiation exposure on patients undergoing fluoroscopy-guided epidural blood patch would be minimal. 

## Conclusions

We successfully performed three fluoroscopy-guided epidural blood patch procedures in the prone position for our patient suffering from SIH. Considering the risk factors for multiple procedures and the practitioner’s stress, we think that the fluoroscopic approach for epidural blood patches is a safe method.
